# Accuracy of genomic selection for alfalfa biomass yield in two full-sib populations

**DOI:** 10.3389/fpls.2022.1037272

**Published:** 2022-10-28

**Authors:** Xiaofan He, Fan Zhang, Fei He, Yuhua Shen, Long-Xi Yu, Tiejun Zhang, Junmei Kang

**Affiliations:** ^1^ School of Grassland Science, Beijing Forestry University, Beijing, China; ^2^ Institute of Animal Science, Chinese Academy of Agricultural Sciences, Beijing, China; ^3^ College of Chemistry and Life Sciences, Chifeng University, Chifeng, China; ^4^ Plant Germplasm Introduction and Testing Research, United States Department of Agriculture-Agricultural Research Service, Prosser, WA, United States

**Keywords:** *Medicago sativa*, genomic selection, full-sib populations, biomass yield, single nucleotide polymorphism

## Abstract

Alfalfa (*Medicago sativa*) is one of the most important leguminous forages, widely planted in temperate and subtropical regions. As a homozygous tetraploid, its complex genetic background limits genetic improvement of biomass yield attributes through conventional breeding methods. Genomic selection (GS) could improve breeding efficiency by using high-density molecular markers that cover the whole genome to assess genomic breeding values. In this study, two full-sib F_1_ populations, consisting of 149 and 392 individual plants (P149 and P392), were constructed using parents with differences in yield traits, and the yield traits of the F_1_ populations were measured for several years in multiple environments. Comparisons of individual yields were greatly affected by environments, and the best linear unbiased prediction (BLUP) could accurately represent the original yield data. The two hybrid F_1_ populations were genotyped using GBS and RAD-seq techniques, respectively, and 47,367 and 161,170 SNP markers were identified. To develop yield prediction models for a single location and across locations, genotypic and phenotypic data from alfalfa yields in multiple environments were combined with various prediction models. The prediction accuracies of the F_1_ population, including 149 individuals, were 0.11 to 0.70, and those of the F_1_ population, consisting of 392 individuals, were 0.14 to 0.67. The BayesC and RF models had the highest average prediction accuracy of 0.60 for two F_1_ populations. The accuracy of the prediction models for P392 was higher than that of P149. By analyzing multiple prediction models, moderate prediction accuracies are obtained, although accuracies will likely decline across multiple locations. Our study provided evidence that GS can accelerate the improvement of alfalfa yield traits.

## Introduction

Alfalfa (*Medicago sativa*) is a perennial leguminous forage with a high yield, high forage value, and wide adaptability and is known as the “Queen of Forages”. Yield is one of the essential traits of alfalfa and is the primary goal of breeding. Yield is a quantitative trait controlled by multiple genes and influenced by the environment (Annicchiarico, 2015). Alfalfa is a homozygous tetraploid, has a complex genetic background, and suffers from severe inbreeding depression and non-additive inheritance, thus limiting the process of alfalfa breeding (Bingham et al., 1994; Annicchiarico et al., 2010). In the past decades, yield improvement has been achieved by increasing the tolerance of alfalfa to stress, so this method of yield improvement only works when stressed (Lamb et al., 2006). Conventional yield breeding has stagnated in recent years, and the narrow heritability for alfalfa yield ranges from 0.15 to 0.30. Low breeding values, long selection cycles, high costs, and low efficiency limit the improvement of alfalfa yield, which requires breeders to use new methods instead of conventional phenotypic selection to improve breeding efficiency (Annicchiarico, 2015).

Genomic selection (GS) is a new approach to select candidate individuals at an early stage or in an off-season nursery or greenhouse based on the prediction of breeding values, which can accelerate genetic gain by capturing genetic information through high-density SNP markers (Lande and Thompson, 1990). The GS study is divided into two main parts, marker development and model prediction. With the development of sequencing technology, there have been various strategies for developing SNP markers. Li et al. identified millions of SNPs in alfalfa by transcriptome sequencing, some of which have been used to develop an Illumina Infinium SNP array containing approximately 10,000 SNP markers (Li et al., 2014). With the rapid development of reduced-representation genome sequencing (RRGS) technology, low-cost RRGS results in SNP markers sufficient to cover the entire genome, of which genotyping-by-sequencing (GBS) and restriction site-associated DNA sequence (RAD-seq) are the two most common methods for RRGS. Based on this, GBS can sequence multiple individuals of a species with known genome sequences and perform differential analyses of individuals or populations (Wallace and Mitchell, 2017). The RAD-seq identifies specific restriction endonuclease sites. Hybridizing RAD tags to DNA microarrays allows simultaneous screening of thousands of polymorphic markers and can be used for bulk genotyping of populations (Baird et al., 2008). These sequencing methods offer the opportunity to obtain high-density and high-quality SNP molecular markers. And next-generation sequencing technology largely increases the efficiency of the determination of the exact genotype of an alfalfa individual (Stritzler et al., 2022). The second step is the prediction of the model, which involves phenotypic and genotypic data from the training population to examine the effects of all SNP markers and develop prediction models to predict GS. A test population is also required to validate the accuracy of the prediction model. Individual genetic strengths were estimated directly as the best linear unbiased prediction (BLUP) (Russell et al., 1997). The genomic best linear unbiased prediction (gBLUP) models for individual-based prediction, the ridge-regression best linear unbiased prediction (rrBLUP), and Bayesian models for SNP benefit value-based prediction, support vector machines (SVM) and random forest (RF), two machine learning methods, are the main approaches currently used to perform GS (Corinna and Vladimir, 1995; Meuwissen et al., 2001; Leo, 2001; VanRaden, 2008; Park and Casella, 2008; Endelman, 2011; Habier et al., 2011). These methods have to be selected according to different populations and different heritabilities of traits. Its prediction accuracy finally reveals the best model. Based on the constructed GS prediction equations, researchers estimate the genomic breeding value (GEBV) of individuals and select candidates early in the nursery or greenhouse, thus improving breeding efficiency.

GS breeding methods have been used for many plants, such as barley, maize, and wheat (Mendes and de Souza, 2016; Schmidt et al., 2016; Juliana et al., 2022). As alfalfa is a perennial forage, the collection of multiple harvest yield data for the purpose of evaluating breeding values is required throughout the breeding process. Furthermore, GS can significantly reduce breeding years to 3–4 years of phenotypic selection if breeding values can be assessed based on genotypes at the seedling stage. Li et al. (2015) used the GS breeding method to make single-location, cross-location, and cross-generation predictions in a comprehensive breeding population of alfalfa. Furthermore, their single-location prediction accuracy was 0.43 to 0.66. The accuracy of cross-generation prediction could also reach 0.40. The GS selection efficiency was higher than the phenotypic selection, which indicates that the GS breeding method could improve the alfalfa breeding efficiency (Li et al., 2015). Creating new germplasm with excellent integrative traits by artificial hybridization is a standard method for alfalfa breeding. GS modeling studies have not been reported for this breeding population. Furthermore, in GS modeling studies, population type, population size, and the number of SNP markers dramatically influence the accuracy of GS prediction, and different GS prediction models can affect prediction accuracy (Paolo et al., 2015). Recent developments in genotyping and prediction models have made it possible to investigate GS in alfalfa breeding populations.

The present study evaluated two full-sib populations of 149 and 392 progeny lines segregating for yield-related traits in three field trials over seven years. The two F_1_ populations were then genotyped using GBS and RAD-seq to obtain SNP markers covering the entire genome. The number of SNP markers from the two library construction methods was compared, and eight GS prediction models were established to analyze the effects of population size and SNP markers on the accuracy of genome prediction.

## Materials and methods

### Plant materials and experimental design

**Figure 1:**
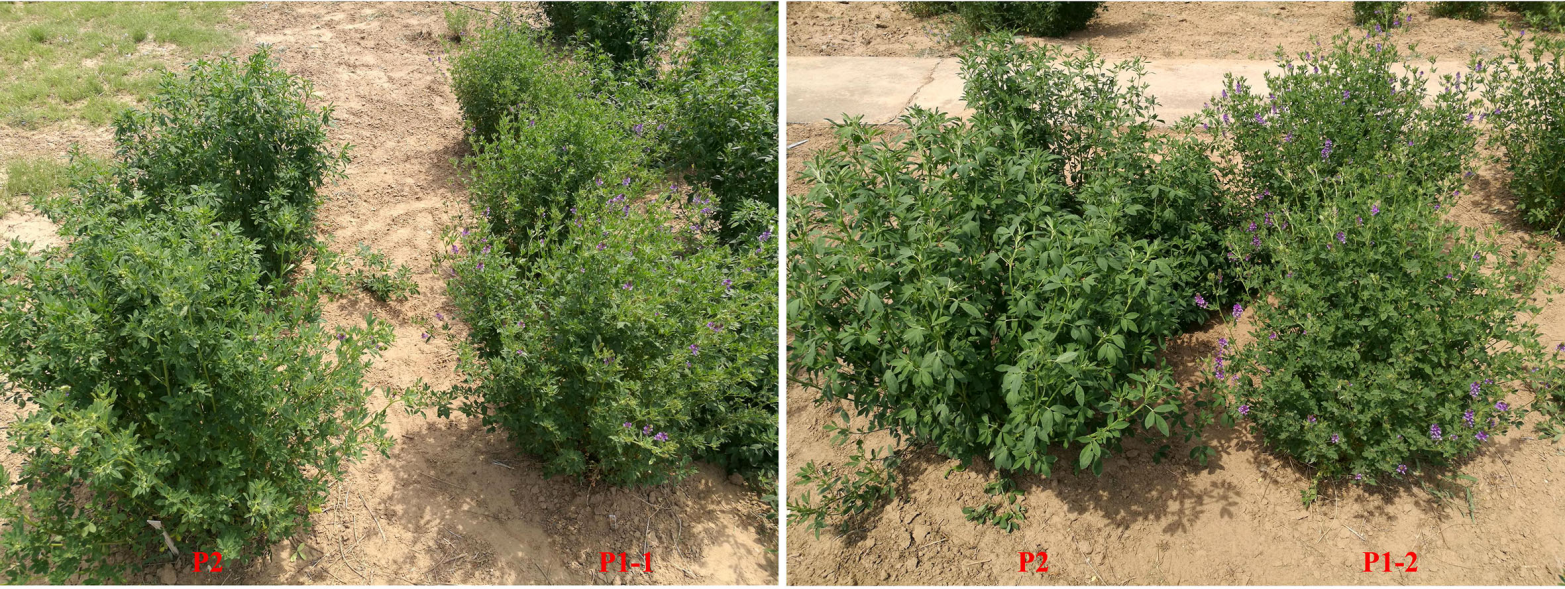
The phenotypic difference between the parents P1-1, P1-2 and P2 for flowering time.

**Figure 2:**
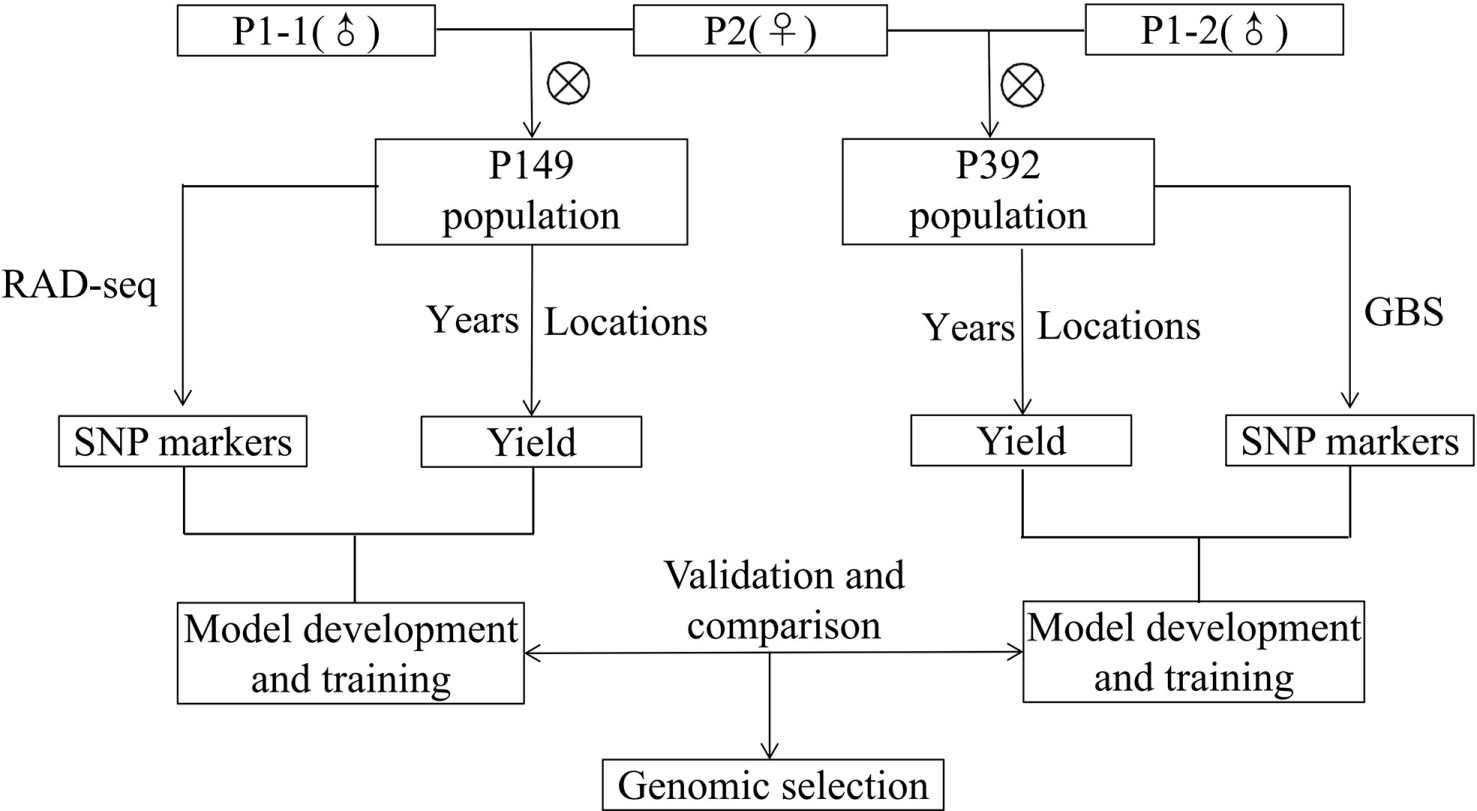
Schematic diagram of training, validation and comparison for genomic selection of alfalfa in full-sib populations.

Population development and field management were provided in a previous study (Zhang et al., 2019; Zhang et al., 2020). Due to its excellent forage yield, large leaves, high stem/leaf ratio, and good persistence of the yield, the same maternal parent, P2, was derived from cultivar Zhongmu No. 1 (CF0032020). Two separate P1 paternal parents (P1-1 and P1-2) with low yield but early flowering time were chosen from a local cultivar, Cangzhou (CF000735, **Figure 1**). The first population, which was designated as P149, included 149 F_1_ individuals, whereas the second population, which was designated as P392, included 392 F_1_ individuals (**Figure 2**). From 2014 to 2019, in Tongzhou and Langfang, eight environments for P149 were sampled for phenotyping. From 2016 to 2020, phenotypic data of P392 from eight environments were also recorded at Langfang and Changping. Harvest dates varied from year to year and depended on the location (**Table 1**).

**Table 1:** Biomass yield harvested by the two populations.

Population	Location	Year	Harvests	Max	Min	Ave.	SE	CV	H^2^
P149	Tongzhou (TZ), Beijing	2014	2	137.07	4.17	56.19	1.66	0.52	0.83
		2015	4	429.17	12.22	104.77	3.78	0.89	0.66
	Langfang (LF), Hebei	2014	3	650.00	9.48	278.87	5.95	0.53	0.44
		2015	4	270.19	2.80	108.53	2.48	0.49	0.91
		2016	4	690.00	14.00	226.65	5.31	0.58	0.91
		2017	1	448.03	22.83	217.12	8.47	0.48	0.02
		2018	1	644.33	99.60	337.46	11.49	0.42	0.05
		2019	4	489.68	1.75	111.94	4.07	0.90	0.94
P392	Langfang (LF), Hebei	2016	3	710.00	14.00	203.02	3.50	0.59	0.97
		2017	1	625.23	25.75	253.99	7.04	0.55	0.83
		2018	3	953.70	6.63	285.15	4.88	0.59	0.80
		2019	2	728.40	20.00	252.88	4.54	0.50	0.96
	Changping (CP), Beijing	2017	4	514.59	5.18	108.56	1.98	0.72	0.81
		2018	3	605.10	22.70	208.64	2.90	0.48	0.95
		2019	4	691.00	25.00	197.27	2.94	0.59	0.89
		2020	4	560.60	2.23	153.31	2.53	0.65	0.97

### Phenotypic data analysis

Every year, one to four harvests were carried out at each location (**Table 1**). Fresh yield of each plant were evaluate, and random samples of individual were harvested for biomass, weighed while still wet, and dried for five days at 60°C in a forced-air facility. The average dry matter biomass yield was calculated based on the average dry-matter content of each plant.

BLUP was performed to evaluate the phenotypic results collected over the years at various locations using PROC MIXED (Russell et al., 1997).

The following was the random-effects model utilized for BLUP:


$$Y_{ijkh}=m+l_{k}+r_{i(k)}+g_{j}+y_{h}+gl_{jk}+gy_{jh}+gly_{jkh}+e_{ijkh}$$

where *Y_ijkh_* represents the Y for the *j*th genotype in the *i*th replication of the *k*th location in the *h*th year; m represents the grand mean; *r_i(k)_* represents the effect of the *i*th replication nested in the *k*th location; y*_h_* represents the effect of the *h*th year; *g_j_* represents the genetic effect of the *j*th genotype; *gl_jk_* represents the interaction effect of the *j*th genotype and *k*th location. The interaction effect of the *j*th genotype and the *h*th year is denoted by the symbol *gy_jh_*; the interaction effect of the *j*th genotype, the *k*th location, and the *h*th year is denoted by the symbol *gly_jkh_*; and the residual is denoted by the symbol e*_ijkh_*. The grand mean was the only element that was not determined by random effects.

The BLUP estimate was carried out using the lme4 package in R (Bates et al., 2015). Estimated BLUPs were used as observed phenotypic values of total biomass output to make a more accurate GS prediction model.

### DNA isolation, RAD, and GBS library construction

DNA extraction and the development of RAD for P149 and GBS for P392 libraries have been described in previous studies (Zhang et al., 2019; Zhang et al., 2020). The RAD sequences and the raw GBS data were uploaded to the NCBI Sequence Read Archive with the BioProject IDs PRJNA503672 and PRJNA522887.

### Sequence analysis and SNP genotyping

The genome of *M. sativa* ‘Xinjiangdaye’ was used as a reference for GBS sequencing analysis of P392 (Chen S. et al., 2020). TASSEL-GBS was used to call SNP with the default parameter (Glaubitz et al., 2014). SNPs with a 50% missing rate and a MAF of 0.05 were filtered using VCFtools (Danecek et al., 2011).

The sequence data from the RAD-seq for P149 was initially filtered for quality using the Trimmomatic program with default parameters (Bolger et al., 2014). The reads obtained from paired-end sequencing were used in BWA-MEM to map *M. sativa* ‘Xinjiangdaye’ genome, using the program’s default mapping settings (Li and Durbin, 2010). It was achieved with SAMtools by converting the SAM data into BAM files and then sorting the BAM files with the default settings (Li et al., 2009). Both Picard Tools and Genome Analysis ToolKit were used in this study. Picard Tools (http://broadinstitute.github.io/picard/) was utilized to mark duplicate reads, while Genome Analysis ToolKit was utilized to repair indels that can be mistaken for SNPs (Van der Auwera et al., 2013). To identify SNPs, the SAMtools mpileup and VarScan programs were utilized (Koboldt et al., 2012). Furthermore, the SNP data were filtered using VCFtools to have a missing rate of less than 10%, a minor allele frequency greater than 0.05, and a mean read depth greater than 20.

The SNP data from the P149 and P392 populations were uploaded to figshare (http://figshare.com) with the following dios: https://doi.org/10.6084/m9.figshare.21172051.v1 and https://doi.org/10.6084/m9.figshare.21162283.v2.

### Correlation analysis

The relationships between the harvest phenotype value in different years and the BLUP estimate in different locations were determined by correlation analysis.

### Model development and validation for genomic selection prediction

A variety of statistical models have been devised for genomic selection (Heffner et al., 2009; Lorenz et al., 2011). The present study evaluated gBLUP, rrBLUP, four Bayesian models, SVM, and RF. The accuracy of the predictions was determined by calculating the Pearson correlation between the predicted and observed phenotypes. To achieve this, 90% of the individuals were assigned to a training set, while the remaining 10% were assigned to a validation set. The genomic prediction accuracy was estimated as the Pearson correlation (r) between estimated GEBVs and estimated BLUPs of phenotypic values. This cross-validation process was carried out 5000 times, and the accuracy ratings were averaged.

gBLUP involves the construction of a covariate matrix by utilizing individual kinships, followed by predicting the phenotype through utilizing the values of individual species (VanRaden, 2008). The following constitutes the statistical model:


y=Xb+Zu+e,

where y represents the association matrix of the phenotypic vector, X represents the association matrix of the fixed effect coefficient, and b represents the fixed effect; Z represents the association matrix of random additive genetic effects; u represents a random additive genetic effect, also known as an individual genomic breeding value; and e represents a residual effect.

rrBLUP operates under the presumption of a linear mixed additive model, in which each marker is given an effect as a solution to the following equation:


y=m+Zu+e,

where y is the observed value of the phenotype, m represents the intercept, Z represents the marker matrix, and u represents a vector of estimated marker effects (Endelman, 2011).

Models based on Bayesian theory provide prior densities for the marker effects that induce the various forms of shrinkage. The answer is produced using a Gibbs sampling strategy to take samples from the resultant posterior density to solve the problem, as detailed in (Geman and Geman, 1984; George and Edward, 2012). In this study, we evaluated the phenotypic prediction capabilities of four different Bayesian models: (i) BayesA (Meuwissen et al., 2001), (ii) BayesB (Habier et al., 2011), (iii) BayesC (Habier et al., 2011), and (iv) Bayesian Ridge regression (Park and Casella, 2008). Using the following settings, the Bayesian models were examined using the BGLR package of the R programming language (Perez and de Los Campos, 2014). The number of iterations equals 5000, while the burnin threshold is set at 1500.

RF uses the bootstrap resampling method to extract multiple samples from the original sample, model a decision tree for each bootstrap sample, combine the predictions of multiple decision trees, and obtain the final prediction result through voting (Leo, 2001). SVM is a model for binary classification, and its learning algorithm is an optimization algorithm for solving convex quadratic programming (Corinna and Vladimir, 1995).

## Results

### Phenotypic analysis

In the two breeding populations, the coefficient of the variation of annual yield ranged from 0.42 to 0.90 (**Table 1**), indicating that the yields of the breeding individuals were quite different. In the P149 population, the broad-sense heritability (H^2^) of LF_2017 and LF_2018 was only 0.02 and 0.05, indicating that the yield in these two years was greatly affected by the environment (**Table 1**).

**Figure 3:**
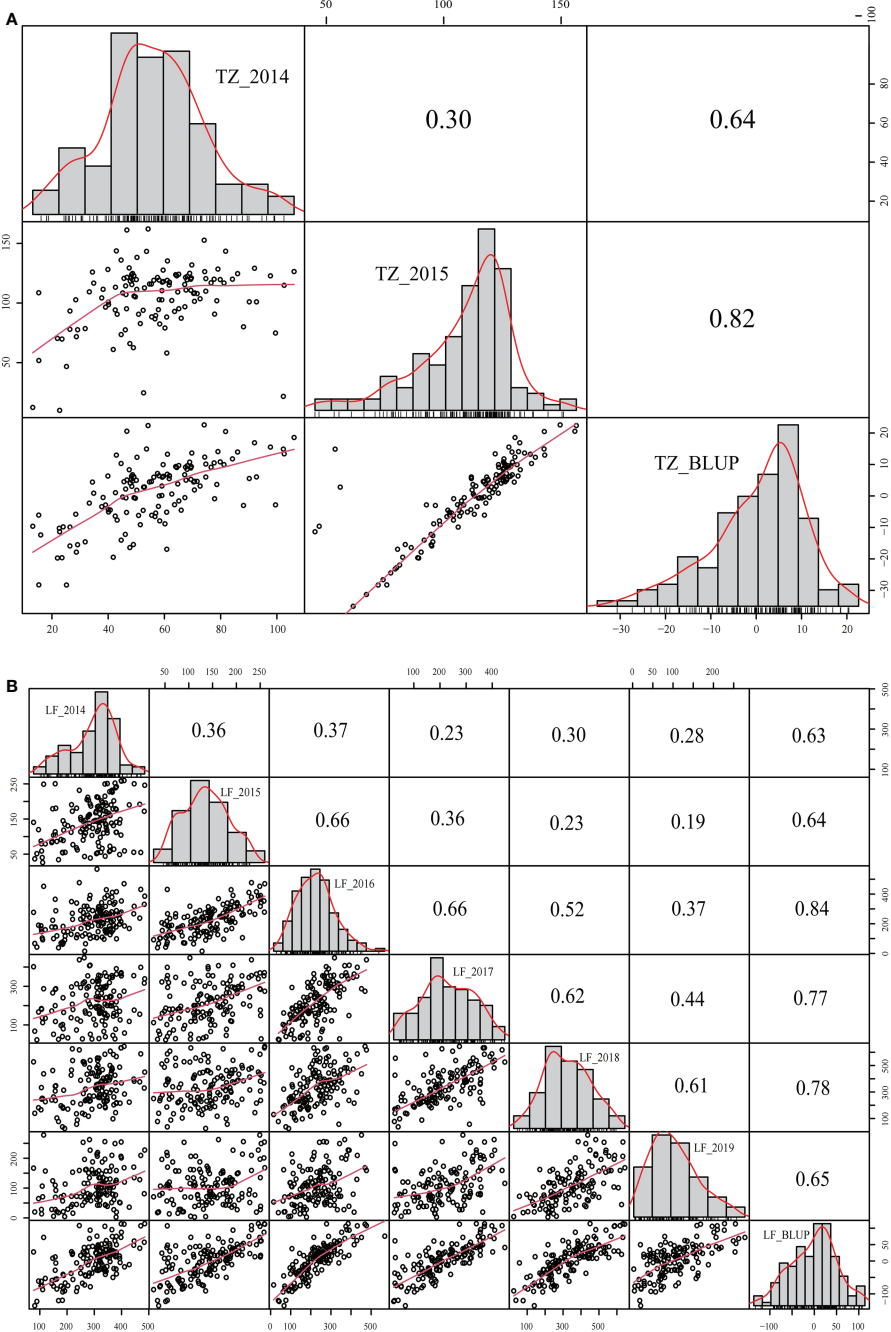
Correlation analysis of different years in the P149 population. **(A)** BLUP was calculated based on years in Tongzhou, Beijing. **(B)** BLUP was calculated based on years in Langfang, Hebei. BLUP, best linear unbiased prediction.

**Figure 4:**
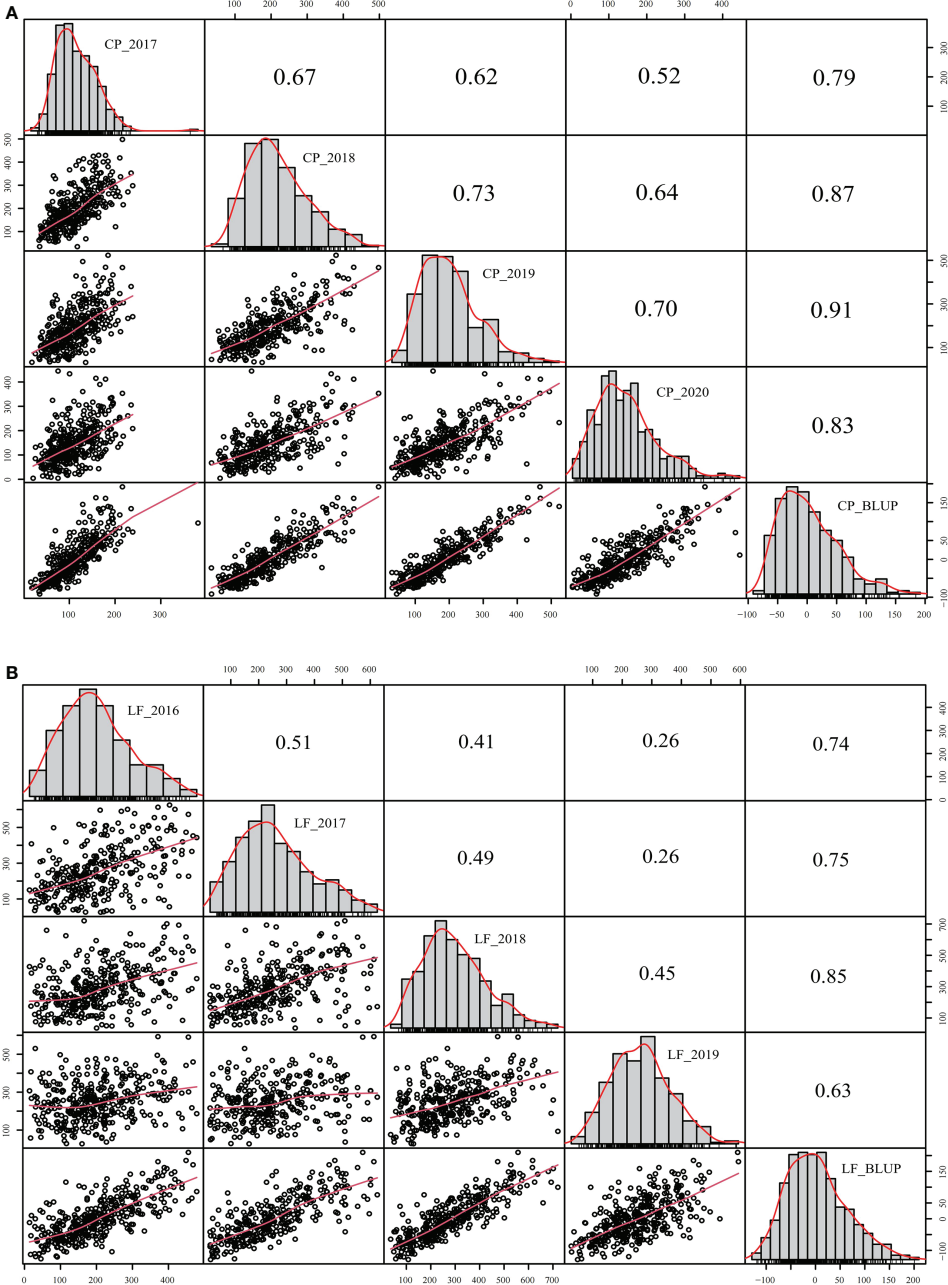
Correlation analysis of different years in the P392 population. **(A)** BLUP was calculated based on years in Changping, Beijing. **(B)** BLUP was calculated based on years in Langfang, Hebei. BLUP, best linear unbiased prediction.

The yield was evaluated by employing the BLUP. Correlation analysis was performed between different years of the two breeding populations using the mean value of the annual yield data and BLUP (**Figures 3**, **4**). The correlations of the yields in different years ranged from 0.19 to 0.73, indicating that the comparisons of the individual yields in different years were quite different. For the yields of P149 and P392, the correlation coefficients between BLUP and each year ranged from 0.63 to 0.91. Overall, the correlation coefficients between BLUP and each year were higher than the values between different years, the only exception being in 2015 and 2016, when the P149 population gave the higher correlation coefficients, 0.66 rather than 0.64.

**Figure 5:**
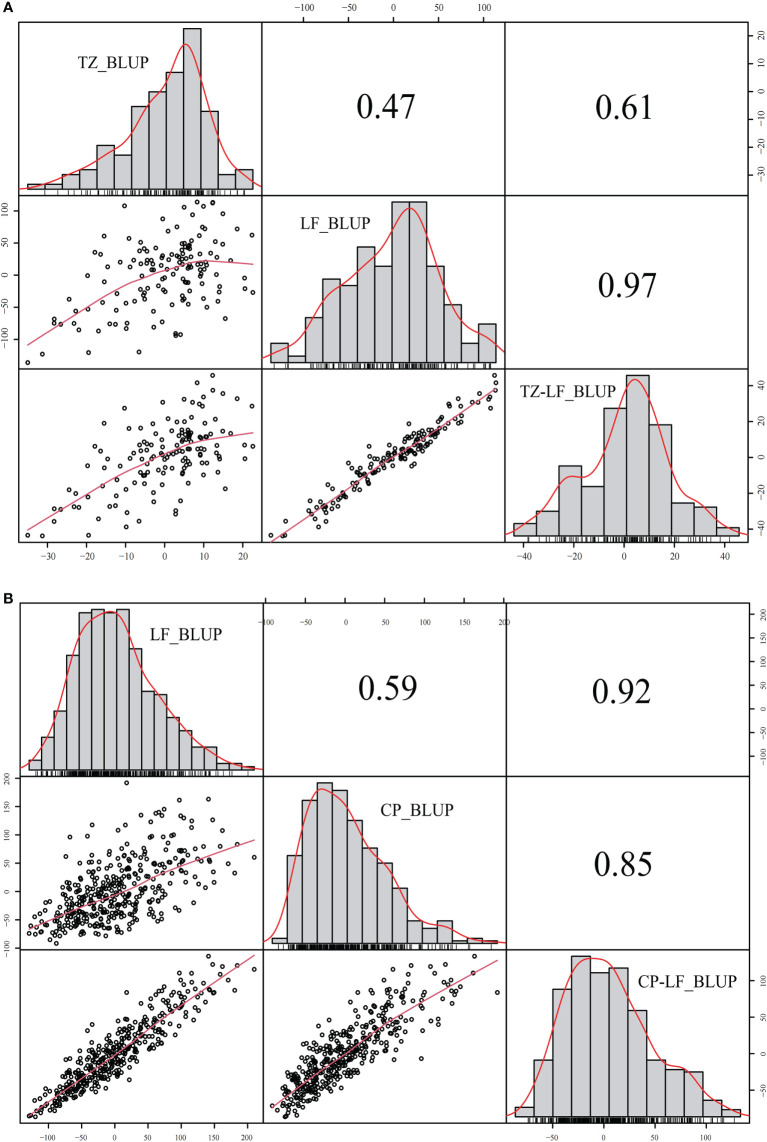
Correlation analysis of different locations in the P149 and P392 populations. **(A)** BLUP was calculated based on locations in the P149 population. **(B)** BLUP was calculated based on locations in the P392 population. Abbreviation: BLUP, best linear unbiased prediction.

Between different locations, a correlation analysis was performed using the mean and BLUP values, and the coefficients for P149 and P392 were 0.47 and 0.59, respectively, indicating a significant difference in yields between different locations (**Figure 5**). In the P392 population, the correlation coefficients between two locations and the overall BLUP were 0.92 and 0.85, indicating that the overall BLUP could represent the value of each location (**Figure 5B**). In the P149 population, the correlation coefficients were 0.61 for TZ_BLUP and TZ-LF_BLUP and 0.97 for LF_BLUP and TZ-LF_BLUP (**Figure 5A**), which were consistently greater than 0.60.

**Figure 6:**
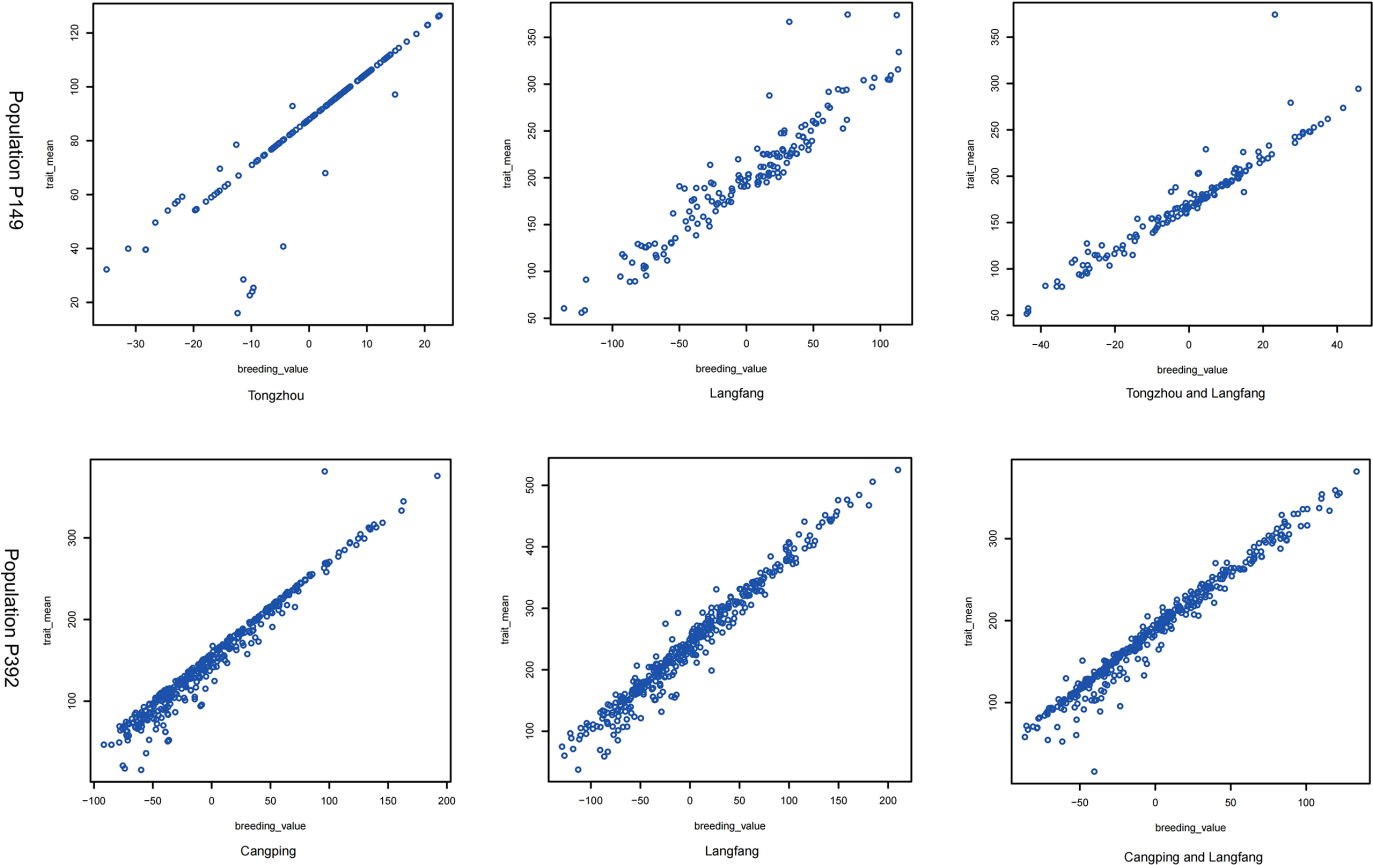
The correlation of BLUP (trait_mean, harvest phenotype value; breeding_value, BLUP phenotype value) in the P149 and P392 populations at a single location and across locations.

Using the BLUP values, the H^2^ of the P149 population at the TZ and LF locations was 0.48 and 0.57, respectively, while the H^2^ of the combined location was only 0.19, which was much lower than the H^2^ of the individual locations (**Table 2**). These results showed significant genetic diversity in the biomass produced by hybrid populations during each harvest and in each environment. On the other hand, the accuracy of the BLUP value and the means of the original data in the two populations was between 0.90 and 0.98, and the correlation coefficients of the combined location were higher than the values of the individual locations, indicating that BLUP could accurately represent the original yield data (**Table 2** and **Figure 6**).

**Table 2:** The result of BLUP in LF and TZ. .

Population	Location^†^	min	max	mean	cor^‡^	H^2^
P149	TZ	-35.05	22.58	0.00	0.90	0.48
	LF	-136.14	113.80	0.00	0.94	0.57
	TZ-LF	-43.80	45.72	0.00	0.95	0.19
P392	LF	-129.11	209.97	0.00	0.98	0.57
	CP	-91.63	191.90	0.00	0.98	0.69
	LF-CP	-86.42	133.38	0.00	0.98	0.48

^†^TZ, Tongzhou, Beijing; LF, Langfang, Hebei; TZ-LF is the total biomass yield at both TZ and LF locations; CP, Changping, Beijing; LF-CP is the total biomass yield at both LF and CP locations. **^‡^** Correlation analysis was performed between the means of phenotype value and BLUP estimate in the two populations.

### Genotyping-by-sequencing genotype data

After quality filtering and processing, a total of 114,943,310,784 sequencing reads were obtained in P149, with an average of 771,431,616 per genotype, ranging from 197,919,225 to 2,813,293,425. A total of 98,802,178,615 sequencing reads were obtained in the P392 population. The average number of reads per genotype was 252,046,374, ranging from 2,637,626 to 779,056,669.

To analyze the sequence reads obtained from two breeding populations coupled with eight chromosomes of *M. sativa* ‘Xinjiangdaye’, we utilized a variety of UNEAK pipelines. After thoroughly examining the readings, we discovered 161,170 SNP markers for the breeding population of P149. After removing markers with missing values greater than 50% of the time, 47,367 SNP markers were retained in the P392 breeding population.

Furthermore, since RAD-seq and GBS sequencing were used in the two populations, we further analyzed the percentage of SNP makers captured jointly and found that the common SNP makers of the two populations were extremely low. A total of 116 shared SNP loci were obtained after the first attempt, which only accounted for 0.07% of the total number of SNPs sequenced by RAD-seq.

### Validation of the P149 population

In P149, the total biomass output was calculated using the BLUP based on six harvests of two years in Tongzhou and seventeen harvests of six years in Langfang. Furthermore, the BLUP values were used as phenotypic data for the GS prediction model. Using the dataset of 161170 SNPs, each one had fewer than 50% of its genotypic values missing.

**Figure 7:**
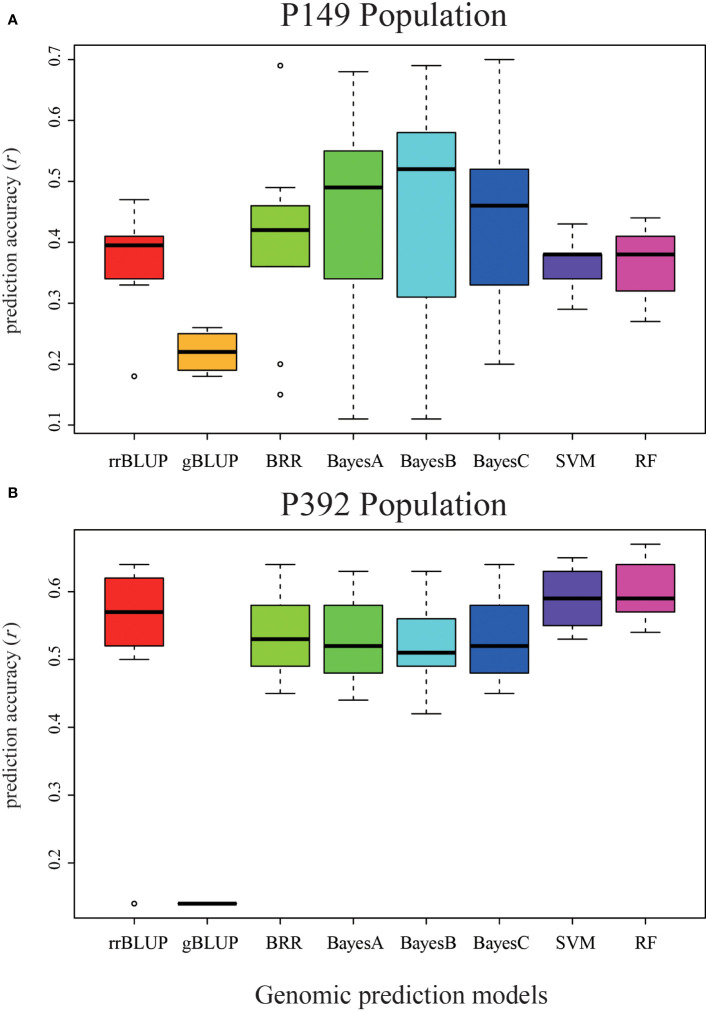
Validation of eight different models in the P149 and P392 populations. **(A)** The genomic prediction accuracy of total biomass yield in the P149 population was validated. **(B)** The genomic prediction accuracy of total biomass yield in the P392 population. rrBLUP, ridge-regression best linear unbiased prediction; gBLUP, genomic best linear unbiased prediction; BRR, Bayesian Ridge regression; BayesA, BayesianA; BayesB, BayesianB; BayesC, BayesianC; SVM, support vector machines; RF, random forest.

We used eight GS models to estimate the accuracy of genomic prediction of single location and across locations, and the accuracy of these models ranged from 0.11 to 0.70. The BayesC model had the highest prediction accuracy of 0.70 in Tongzhou, the BayesB model had the highest prediction accuracy of 0.58 in Langfang, and the BayesB model had the highest prediction accuracy of 0.55 for total biomass at both sites (**Table 3** and **Figure 7A**). Among these eight models, the gBLUP model predicted the lowest average accuracy of 0.22, while the BayesB and BayesC models predicted the highest average accuracy of 0.43.

**Table 3:** The genomic prediction accuracy of total biomass yield in the P149 population validated within and across locations.

		Validation^†^			
Model	Estimation	TZ	LF	TZ-LF	Mean
rrBLUP	TZ	0.47	0.33	0.34	0.39
	LF	0.38	0.40	0.41	
	TZ-LF	0.39	0.40	0.41	
gBLUP	TZ	0.18	0.18	0.19	0.22
	LF	0.19	0.25	0.26	
	TZ-LF	0.19	0.25	0.25	
BRR	TZ	0.69	0.15	0.20	0.40
	LF	0.36	0.46	0.49	
	TZ-LF	0.42	0.40	0.42	
Bayes A	TZ	0.68	0.11	0.17	0.43
	LF	0.34	0.55	0.58	
	TZ-LF	0.40	0.49	0.53	
Bayes B	TZ	0.69	0.11	0.17	0.43
	LF	0.31	0.58	0.60	
	TZ-LF	0.37	0.52	0.55	
Bayes C	TZ	0.70	0.20	0.23	0.43
	LF	0.33	0.52	0.55	
	TZ-LF	0.39	0.46	0.49	
SVM	TZ	0.44	0.29	0.32	0.36
	LF	0.34	0.38	0.39	
	TZ-LF	0.35	0.38	0.38	
RF	TZ	0.44	0.27	0.29	0.36
	LF	0.42	0.36	0.38	
	TZ-LF	0.41	0.32	0.38	

^†^TZ, Tongzhou, Beijing; LF, Langfang, Hebei; TZ-LF is the total biomass yield at both TZ and LF locations.

It should be noted that the accuracy of the predictions of the TZ-LF and TZ-TZ-LF models for the four Bayesian models was very low in P149, and to explore the reason for this, we subsequently performed the validation of the GS model following the same method using biomass data for two years (2014 and 2015) from Tongzhou and Langfang. The model prediction accuracies ranged from 0.17 to 0.70, with single-location predictions from TZ, LF, and TZ-LF having the highest accuracy of the BayesC model at 0.70, 0.57, and 0.65, respectively. The gBLUP model had the lowest prediction accuracy of 0.19, and the BayesC model had the highest prediction accuracy of 0.60 among the eight models (**Table 4**).

**Table 4:** The genomic prediction accuracy for two years (2014 and 2015) of biomass yield in the P149 population validated within and across locations.

		Validation^†^			
Model	Estimation†	TZ	LF	TZ-LF	Mean
rrBLUP	TZ	0.47	0.42	0.41	0.45
	LF	0.50	0.44	0.43	
	TZ-LF	0.49	0.43	0.43	
gBLUP	TZ	0.18	0.17	0.18	0.19
	LF	0.20	0.19	0.21	
	TZ-LF	0.19	0.19	0.20	
BRR	TZ	0.69	0.53	0.62	0.58
	LF	0.54	0.54	0.63	
	TZ-LF	0.55	0.53	0.62	
Bayes A	TZ	0.68	0.54	0.63	0.58
	LF	0.50	0.55	0.64	
	TZ-LF	0.53	0.55	0.64	
Bayes B	TZ	0.69	0.52	0.62	0.58
	LF	0.51	0.53	0.62	
	TZ-LF	0.54	0.53	0.63	
Bayes C	TZ	0.70	0.57	0.65	0.60
	LF	0.52	0.57	0.65	
	TZ-LF	0.54	0.57	0.65	
SVM	TZ	0.44	0.37	0.38	0.39
	LF	0.42	0.37	0.37	
	TZ-LF	0.44	0.38	0.37	
RF	TZ	0.44	0.36	0.36	0.41
	LF	0.53	0.35	0.35	
	TZ-LF	0.47	0.42	0.38	

^†^TZ, Tongzhou, Beijing; LF, Langfang, Hebei; TZ-LF is the total biomass yield at both TZ and LF locations.

### Validation within the P392 population

For the P392 population, the total biomass yield was calculated by applying BLUP, using harvest yield data for four years of nine harvests at Langfang and four years of 15 harvests at Changping, and the BLUP values were used for the GS prediction model. When using the data set consisting of 47,367 SNPs, each had fewer than 50% of their genotypic values missing.

The highest accuracy of the total biomass yield was predicted by the RF model in Langfang, two locations, and the rrBLUP model in Changping, with 0.56, 0.67, and 0.61, respectively. The overall prediction accuracy of the eight models ranged from 0.14 to 0.67 (**Table 5**). The gBLUP model predicted the lowest average accuracy of 0.14, and the RF model predicted the highest average accuracy of 0.60 (**Table 5** and **Figure 7B**). The highest average accuracy of 0.60 was achieved by the RF model.

**Table 5:** The genomic prediction accuracy of total biomass yield in the P392 population validated within and across locations.

		Validation^†^			
Model	Estimation†	LF	CP	LF-CP	Mean
rrBLUP	LF	0.52	0.55	0.62	0.58
	CP	0.50	0.61	0.62	
	LF-CP	0.53	0.59	0.64	
gBLUP	LF	0.14	0.14	0.14	0.14
	CP	0.14	0.14	0.14	
	LF-CP	0.14	0.14	0.14	
BRR	LF	0.46	0.45	0.53	0.53
	CP	0.53	0.58	0.64	
	LF-CP	0.49	0.53	0.58	
Bayes A	LF	0.46	0.44	0.52	0.53
	CP	0.51	0.58	0.63	
	LF-CP	0.48	0.53	0.58	
Bayes B	LF	0.45	0.42	0.51	0.52
	CP	0.52	0.56	0.63	
	LF-CP	0.49	0.50	0.57	
Bayes C	LF	0.45	0.45	0.52	0.53
	CP	0.52	0.58	0.64	
	LF-CP	0.48	0.53	0.58	
SVM	LF	0.54	0.58	0.63	0.59
	CP	0.53	0.60	0.65	
	LF-CP	0.55	0.59	0.65	
RF	LF	0.56	0.59	0.64	0.60
	CP	0.57	0.59	0.67	
	LF-CP	0.54	0.59	0.67	

^†^LF, Langfang, Hebei; CP, Changping, Beijing; LF-CP is the total biomass yield at both TZ and LF locations.

### Validation based on the number of markers

**Figure 8:**
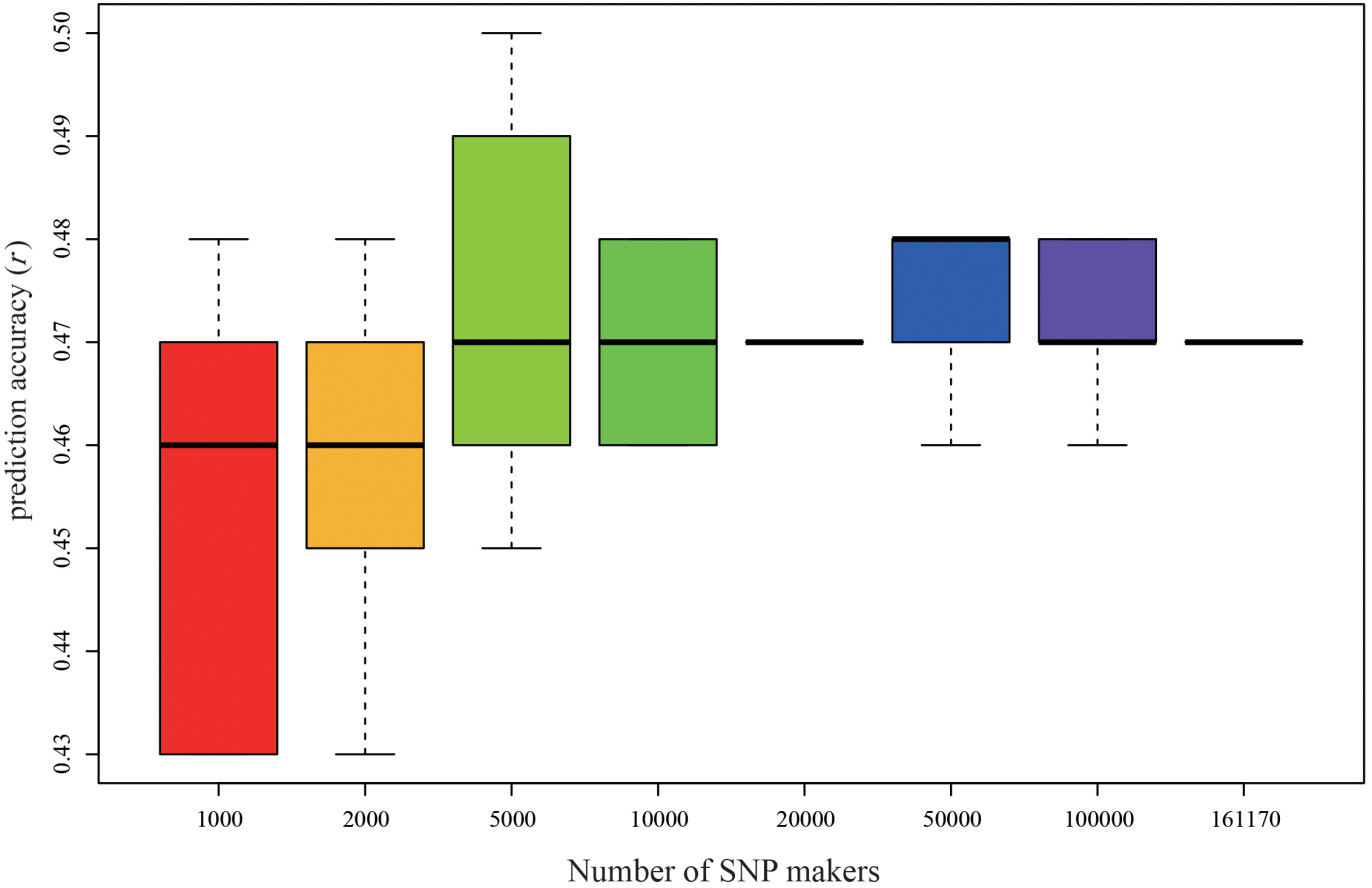
Genomic prediction accuracy of biomass yield at Tongzhou in P149 using different levels of marker datasets by rrBLUP. rrBLUP, ridge-regression best linear unbiased prediction.

In the P149 population, we randomly selected different SNP markers for the GS analysis of the Tongzhou yield data using the rrBLUP model, with five replications for each number of SNP markers. When the number of SNP markers is 1000, the prediction accuracy is at least 0.45, and when the number of SNP markers is more than 5000, the prediction accuracy is 0.47 in all cases (**Figure 8**).

## Discussion

### Genotyping-by-sequencing and restriction-site-associated DNA sequencing

GBS and RAD-Seq are two of the most common simplified genome sequencing technologies that have been used to detect SNP loci in the genomes of many plants. The GBS and RAD-seq methods employ methylation-sensitive enzymes to trim genomic sequences and decrease redundancy. However, the sensitivity enzymes and sequencing methodologies used by the two systems are distinct (Zhang et al., 2019). In this study, we performed RAD-Seq and GBS on the P149 and P392 populations, respectively, to explore the effect of the number of SNP markers on the prediction accuracy of the GS model.

GBS simplifies the library building step and is a cost-effective method for SNP typing, but the number of SNPs obtained is significantly lower than RAD-Seq (Davey et al., 2011). In the P149 population, we identified 161,170 SNP loci in tetraploid alfalfa that could be used for genotyping using RAD-Seq, and in the P392 population, we obtained 47,367 SNP loci that could be used for genotyping using GBS, which was only 29.4% of the RAD-Seq method. It showed that the RAD-Seq sequencing method obtained significantly more SNPs than the GBS method.

Due to the covariance between the genomes of *M. truncatula* and *M. sativa*, many previous studies have used the *M. truncatula* genome as a reference for genotype data analysis, although *M. truncatula* is self-pollinated and diploid (Chen H. et al., 2020). The most significant challenge we encounter with alfalfa (as we do with other allogamous polysomic polyploids) is the presence of heterozygosity. Multiple readings are necessary to get an accurate determination of whether a genotype is heterozygous or homozygous. The availability of the whole genome sequence of *M. sativa* ‘Xinjiangdaye’ (Chen S. et al., 2020) allows us to compare the simplified genome sequence with the entire alfalfa genome, eliminating the dilemma of using *M. truncatula*. In this study, we aligned the simplified genome sequences with the reference genome of alfalfa and obtained 161,170 and 47,367 SNP markers that increased by 42% and 65% compared to the previous analysis using the *M. truncatula* genome (Chen S. et al., 2020). Increasing the number of SNP loci can significantly increase the power of association mapping and genomic prediction.

### Genomic prediction accuracy based on the number of markers

As the number of SNPs increased, the accuracy of the GS model prediction also increased, but the accuracy of the predictions eventually reached a plateau. Predictions using 35,000 GBS SNP markers with up to 80% missing data were more accurate than predictions using 2000 diversity array technology (DArT) markers with 2% missing data in barley (*Triticum aestivum* L.) (Poland et al., 2012). For increasing marker counts and avoiding bias caused by DArT markers, GBS was mainly considered for increasing the accuracy of genomic prediction (Lu et al., 2013), thus improving the accuracy of genomic prediction. In alfalfa, the average accuracy of GS increased as the number of markers increased until the amount of missing data per marker exceeded the limitation of 70 to 80%, as a higher imputation error may be caused by higher missing values (Li et al., 2015). In this study, the number of SNP markers increased from 1000 to 161,170, but the prediction accuracy did not improve significantly and fluctuated around 0.47 when the number of SNP markers exceeded 5000.

Since simplified sequencing can only cover about 3% of the genomic regions, GBS and RAD-seq can capture a few genomic regions together, and thus the number of common SNPs that can be detected is also small. In this study, after several attempts, we found that the common SNP loci only represented 0.07% of the total SNPs sequenced by RAD-seq, which was close to the theoretical value of 0.09%. Therefore, it was impossible to combine the data from the two sequencing results for the integration validation of the yield model in two populations, P149 and P392, at a later stage. In order to improve the applicability of the yield model, representative loci with comprehensive coverage and uniformity should be selected to design genotype detection chips and establish a yield prediction model based on a uniform genotype data matrix. Then, when genotype analysis is done on the population to be tested, the yield model created in the first step can be used to predict the yield of the population.

### Genomic prediction accuracy based on the model developed

GS breeding methods have been used for many plants, such as winter barley, with prediction accuracies of 0.80 for malt quality, 0.72 for maize yield, and 0.94 for oil palm fruit yield (Cros et al., 2015; Mendes and de Souza, 2016; Schmidt et al., 2016). In this study, two half-sib populations were harvested at three different locations, and multi-year yield data were collected. We obtained 0.70 accuracy using the BayesC model in the P149 population, indicating that sufficient accuracy can be predicted for complex traits such as alfalfa yield in half-sib alfalfa populations.

Different GS models resulted in different accuracies in marker assumptions and treatment effects. This study used the gBLUP model, which predicts on an individual basis, and the rrBLUP, BRR, BayseA, BayesB, and BayesC models, which predict on an SNP benefit value basis. The results showed that gBLUP was less effective than the other models, which indicates that predicting yield using individuals is not ideal for alfalfa. Moreover, prediction based on SNP effect values could lead to better prediction accuracy.

The worst prediction accuracy of the gBLUP model may be related to the fact that the gBLUP model is based on individual prediction, with a heritability of yield traits of approximately 19% and approximately 80% of phenotypic variation that cannot be predicted by individual effects, resulting in lower prediction accuracy. Furthermore, the covariance used in this study was replaced by the principal component analysis (PCA) results using TASSEL to calculate genotype data. However, the covariance between individuals for a specific trait is determined by the association at the causal loci rather than the association across the entire genome (Endelman and Jannink, 2012). This is because causal loci are more likely to be inherited. Recent research has shown that even for complicated characteristics with very high marker density, the connection at the whole genome level can only poorly approximate the causative locus relationship for individuals that are very distantly related (Hill and Weir, 2011; de Los Campos et al., 2013). This is why the prediction accuracy of the gBLUP model is lower than that of other methods. Because of the low prediction accuracy of the gBLUP model, we will not discuss this model afterwards.

### Genomic prediction accuracy based on location

For the GS models used in the P149 population, we established four Bayesian models, among which the TZ-LF and TZ-TZ-LF models predicted very low accuracy. However, the prediction accuracy of the TZ single-location was all above 0.67. This is probably because Tongzhou had only two years of data for six harvests. Because of the disparity in the volume of the data, a GS model was developed that failed to capture the majority of biomass yield genes across ages or growth seasons, and as a result, it had an inaccurate forecast. We then used two years of data from Tongzhou and Langfang (2014 and 2015). When the GS model was rebuilt, the accuracy of its predictions increased from 0.11–0.33 to 0.42–0.57, and the accuracy of both the local and cross-location predictions increased. This showed us that the stubble numbers of the modeling and validation populations should not be too different when conducting GS breeding and that the quality of biomass data can significantly impact the accuracy of the GS model prediction.

In addition, population size also affects the prediction accuracy of GS models. Generally, the larger the breeding population, the higher the accuracy of GS prediction and the higher the cost. We constructed two populations including different individuals to investigate the effect of the population size on the accuracy of the GS prediction. The seven models showed high prediction accuracy for the P392 population (0.42–0.67). In conclusion, when developing the alfalfa GS model, we should establish as large a breeding population as we can afford.

### Implications for alfalfa breeding

In this study, the prediction accuracy of large breeding populations was higher than that of small breeding populations. The prediction accuracy of total biomass was high, and high accuracy was also observed in cross-location prediction. Between different models the prediction accuracy varied greatly. It is also surprising that when the number of SNP makers increases, the prediction accuracy does not increase significantly, which will be our next research direction. This study shows that GS has a high potential to accelerate alfalfa breeding.

## Data availability statement

The datasets presented in this study can be found in online repositories. The names of the repository/repositories and accession number(s) can be found in the article/Supplementary Material.

## Author contributions

TZ and JK designed and supervised the project. XH performed bioinformatic analysis and drafted the manuscript. FZ and FH performed experiments and participated in the bioinformatic analysis. YS collected the samples, maintained the growth of the experiment materials, and performed DNA extraction. L-XY revised the manuscript and participated in the interpretation of the data. TZ and JK contributed equally as corresponding authors. All authors contributed to the article and approved the submitted version.

## Funding

This work was supported by the Breeding and Industrialization Demonstration of New High-quality Alfalfa Varieties (No. 2022JBGS0020), National Natural Science Foundation of China (No. 31772656), and China Forage and Grass Research System (CARS-34).

## Conflict of interest

The authors declare that the research was conducted in the absence of any commercial or financial relationships that could be construed as a potential conflict of interest.

## Publisher’s note

All claims expressed in this article are solely those of the authors and do not necessarily represent those of their affiliated organizations, or those of the publisher, the editors and the reviewers. Any product that may be evaluated in this article, or claim that may be made by its manufacturer, is not guaranteed or endorsed by the publisher.
